# Sustainable Synthesis of Copper Nanoparticles in 3D-Printed Microfluidic Devices: Effect of pH and Mixing Kinetics on Physicochemical Properties

**DOI:** 10.3390/nano16120772

**Published:** 2026-06-19

**Authors:** Nicolás Ateaga, Dreidy Vásquez, Juan Carlos González, Antonio Molina, Valentina Díaz, Rodrigo Ortiz-Soto

**Affiliations:** Escuela de Ingeniería Química, Facultad de Ingeniería, Pontificia Universidad Católica de Valparaíso, Avenida Brasil 2162, Valparaíso 2362807, Chile; nicolas.ateaga@pucv.cl (N.A.); juan.gonzalez.h@pucv.cl (J.C.G.); antonio.molina.p@mail.pucv.cl (A.M.); valentina.diaz.o@mail.pucv.cl (V.D.); rodrigo.ortiz@pucv.cl (R.O.-S.)

**Keywords:** green synthesis, copper nanoparticles, microfluidic devices, 3D printing, L-ascorbic acid

## Abstract

Green synthesis of metal nanoparticles has attracted significant attention due to its sustainability, yet achieving precise control over their physicochemical properties via continuous-flow systems remains a challenge. This study evaluates the sustainable synthesis of copper nanoparticles using 3D-printed microfluidic reactors fabricated via the fused filament technique with glycol-modified polyethylene terephthalate. A systematic experimental design was performed to investigate the effects of the reducing agent concentration, the channel architecture, and the medium pH on particle size and morphology. Fluid dynamics theoretical modeling revealed a laminar flow regime, dominated by advection, where the serpentine geometry successfully induced stable homogeneous mixing. Statistical analysis identified pH as the most critical factor, demonstrating that an alkaline medium of pH 8 combined with a 5:1 reductant-to-precursor ratio optimizes the production of uniformly spherical copper nanoparticles with significantly smaller diameters. Advanced experiments also assessed the influence of flow rates and stabilizer agents on particle size, morphology and purity. These findings validate the integration of additive manufacturing and continuous microfluidics as a robust, low-cost, and eco-friendly platform for the reproducible and scalable production of metallic nanoparticles.

## 1. Introduction

The synthesis of nanomaterials plays a crucial role in advancing technological innovations across various fields, including electronics, medicine, and environmental science. Copper nanoparticles (CuNPs) are particularly significant due to their unique physicochemical properties, such as excellent electrical conductivity, catalytic activity, and antimicrobial effects, which make them highly desirable for applications ranging from electronics to biomedical devices. Comprehensive knowledge of the synthesis and manipulation of CuNPs is essential for optimizing their properties and broadening their technological applicability. In this context, adopting a green synthesis approach for this metal is critical to advancing sustainable development goals within nanotechnology. This strategy minimizes the use of hazardous chemicals and reduces waste generation, strictly aligning with the core principles of green chemistry and environmental sustainability [1].

Diverse studies on green synthesis have demonstrated that it is possible to produce copper nanoparticles using natural extracts from plants, fruit residues and ascorbic acid among others [2]. Among natural reductants, L-ascorbic acid is notable for its dual role as a reducing agent and stabilizer, although its use has not yet become widespread [3]. Chemically, ascorbic acid reduces Cu^2+^ ions to Cu^0^ by donating two electrons, thereby converting to dehydroascorbic acid [3]. This process is highly pH-dependent, as pH modulates the redox potential of the system and, consequently, the kinetics of particle formation [4].

Among the studies using ascorbic acid as a reducing agent, Umer et al. obtained copper nanoparticles of 100 nm with cubic morphology using ethylene glycol as a solvent and PVP40 as a stabilizer; they also synthesized copper particles of 70 nm with deionized water and hypophosphite/oleic acid/NH_3_, all using copper sulfate as a precursor, and 2 nm particles using copper chloride and deionized water with the same reductant as a stabilizer [5]. Other authors obtained copper nanoparticles with particles sizes around 3 nm using PVP as a stabilizer [6], and more than 10 nm using PEG as a stabilizer [7]. The most recent work used copper chloride in different solvents (water, ethylene glycol and diethylene glycol) with a molar ratio of 1:2 and 80 °C for 16 h of the reaction, and they found that the most suitable solvent was EG to obtain sub-10 nm copper particles [4].

An alternative method to batch synthesis is continuous-flow processing, and one interesting example of this process is microfluidics, which enables precise control over reaction conditions. This technology has emerged as a promising platform for the green synthesis of nanoparticles. These devices facilitate the continuous production of nanoparticles with uniform size and shape, enhancing their quality and functionality. Recent advances in microfluidic technology have enabled the precise manipulation of reaction parameters, such as pH, reductant concentration, and residence time, which are critical for determining the morphology, size, and purity of nanoparticles in a short time [8]. This approach had been utilized from 2010 to 2015 for copper nanoparticle synthesis using toxic reagents as reducing agents. Ket et al. used stainless-steel concentric tubes with 1.00 mm ID and 10 mm length at 20 °C and 1840 mL/min for the production of spherical core–shell particles of 2 to 5 nm in size [9]. Ramprassad generated core–shell spheres with 40 to 50 nm size at 120 °C in a “T”-shaped device of stainless steel (SS) at 0.5 mL/min and 2 mm channel [10]. Other studies with “T”-shaped microreactors in PDMS and SS were performed at room temperature, 0.4 and 1.58 mm channels, respectively, at basic pH (10 to 12) with 0.16 to 10 mL/min, obtaining core–shell spherical particles with sub-20 nm size [11,12,13,14]. Meanwhile a “Y”-shaped polytetrafluoroethylene (PTFE) microfluidic device with a 0.13 mm channel operating at a flow rate of 1.67 mL/min produced core–shell and polyhedral forms with sizes between 6.6 and 136 nm [15]. On the other hand, a “U”-shaped type of poly(methyl methacrylate) with a 0.25 mm channel and pH 13 produced core–shell spheres with sizes of 1.21 and 1.96 nm [16]. The most recent study in this area used a soda-lime glass microfluidic with a Y-shaped microchannel followed by a serpentine mixer of seven turns, 260 μm width and 70 μm depth, featuring a cross-sectional area shaped like a semi-sphere. The synthesis was performed with copper chloride dihydrate in ethylene glycol at a molar ratio of 1:10 with L-ascorbic acid at three flow rates: 0.0001 mL/min, 0.02 mL/min and 0.04 mL/min and at 160 °C, obtaining particles sizes between 6 and 3 nm, respectively [17].

All these studies demonstrated the opportunity that microfluidics offers for the synthesis of copper nanoparticles. With the growth of additive manufacturing (AM), this technology has more opportunities. The use of microfluidic reactors (MFRs) fabricated by 3D printing offers several advantages, including flexibility in design, rapid prototyping, and scalability [18,19], making it an ideal approach for low-cost nanoparticle synthesis with higher control of morphology, sizes and distribution. In early studies on the synthesis of metal nanoparticles (MNPs) using AM, Kiston et al. reported the synthesis of 10 nm gold nanoparticles using tetrachloroauric acid (HAuCl_4_) as a precursor and sodium borohydride (NaBH_4_) as a reducing agent in an aqueous medium. In this study, polypropylene MFRs fabricated using the fused filament technique (FFF) were used [20]. Bressan et al. reported the synthesis of silver nanoparticles smaller than 10 nm and gold nanoparticles smaller than 50 nm using silver nitrate (AgNO_3_) and gold (III) chloride (Au_2_Cl_6_) as precursors, respectively, with sodium borohydride (NaBH_4_) as the reducing agent in an aqueous medium. In this study, poly(lactic acid) and poly(methyl methacrylate) MFRs fabricated by FFF were used [21]. The following year, the same authors, Bressan et al., reported the synthesis of silver-coated gold nanoparticles (Au@AgNPs core–shell NPs) smaller than 30 nm using a poly(lactic acid) MFR fabricated by FFF [22]. However, the synthesis of copper nanoparticles with microfluidic devices fabricated by 3D printing has not yet been reported.

Despite significant progress in the synthesis of CuNPs using MFRs, several aspects remain unclear. Previous research has largely ignored the detailed interaction effects between synthesis parameters, such as pH and reductant concentration, on the final properties of CuNPs. In contrast to earlier studies, which focused primarily on individual parameter effects, the comprehensive exploration of parameter interactions is crucial for achieving precise control over nanoparticle characteristics. What remains unclear is the extent to which these interactions influence the scalability and reproducibility of the synthesis process. Addressing this gap is essential for advancing the field of nanomaterial synthesis and for optimizing the production of CuNPs for industrial applications. The development of a systematic approach to investigate these interactions will contribute to the refinement of synthesis methodologies and the enhancement of nanoparticle quality. The aim of this study is to evaluate the effects of pH, reductant concentration, turns of microfluidic reactor, flow rate and stabilizer agents on the morphology, size, and purity of copper nanoparticles synthesized using microfluidic devices fabricated by FFF 3D printing with glycole-modified poly(ethylene terephthalate) (PETG). It is expected that the interaction between pH and reductant concentration plays a significant role in determining the final properties of the nanoparticles. To test this hypothesis, microfluidic devices with Y-shaped and serpentine channels were used to synthesize CuNPs under controlled conditions.

## 2. Materials and Methods

### 2.1. Materials

Copper sulfate pentahydrate (CuSO_4_·5H_2_O, >98%, Vimaroni, Valparaíso, Chile), L-ascorbic acid (C_6_H_8_O_6_, >99%, Sigma-Aldrich, St. Louis, MO, USA), sodium hydroxide (NaOH, >99%, Emsure, Merck KGaA, Darmstadt, Germany), polyvinylpyrrolidone (PVP K30, Huangshan Bonsun Pharmaceutics, Huangshan, China), pure ethanol (C_2_H_5_OH, >99%, Sigma-Aldrich) and distilled water were used for the synthesis of the nanoparticles.

### 2.2. Microfluidic Device Manufactured by 3D Printing

**Figure 1:**
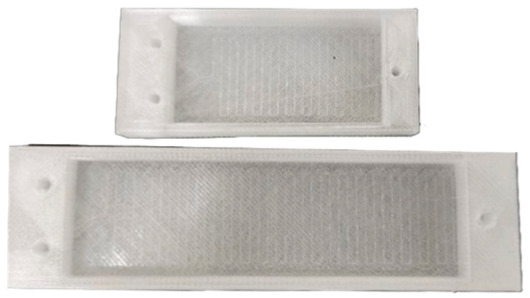
MFRs printed with PETG with 20- and 40-turn serpentine mixing.

The open-source software Flui3D [23] was used to design the microfluidic device. A Y-type inlet was selected to inject the primary reagents into the device, such as the copper ion precursor solution and the reducing agent solution, followed by a set of serpentine mixing channels with a square cross-section. Two devices were considered with 20 and 40 turns and identical cross-sectional dimensions, respectively. In a second step, Blender 5.1 was used to modify the design in order to make visible the mixing section of the chip. Finally, Bambulab A1 was used to print the device with glycole-modified poly(ethelylene terephthalate) (PETG, 1.75 mm, transparent, Todo Toner, Santiago, Chile) chosen for its chemical resistance and mechanical stability. Figure 1 shows 20- and 40-turn 3D-printed microfluidic devices for CuNP syntheses.

### 2.3. Copper Nanoparticles Synthesized with Microfluidic Devices

A first study for the green synthesis of CuNPs was carried on with 20 mL 0.1 M of copper sulfate aqueous solution with 20 mL of L-ascorbic acid aqueous solution at reductant-to-precursor ratio (ratio R/P) of 2:1 and 5:1. Both solutions were introduced in two separated inlets by automated syringe pumps at 0.4 mL/min total flow rate, using the 20- and the 40-turn devices. Then, the nanoparticles were collected from the outlet and separated by decantation, and collected and stored in pure ethanol. In this preliminary study a 2^2^ × 3 experimental design (DoE) was performed with a central point extended to analyze the effect of pH, the reductant-to-precursor ratio and residence time on the morphology and particle size of the CuNPs. Table 1 presents the parameters studied. To study the effect of pH a 1 M of sodium hydroxide was used to change the pH of the L-ascorbic acid aqueous solution. Sample sizes were determined based on preliminary trials to ensure statistical power and reproducibility. Randomization was employed to minimize bias, and blinding was implemented during data analysis to ensure objectivity.

After the analysis of this preliminary study, three parameters were fixed at pH 8, a ratio (R/P) of 5:1 and 20 turns of the serpentine mixer and a second experimental design (2 × 3) was performed to study the effects of flow rate and PVP concentration (0, 10 and 25 g/L) as stabilizing agent on the morphology, particle size, and purity of the CuNPs (Table 2):

### 2.4. Copper Nanoparticle Characterization

The nanoparticles were then characterized using scanning electron microscopy (FESEM, Thermo Scientific Quattro S, Waltham, MA, USA) to assess morphology and particle size. Energy-dispersive X-ray spectroscopy (EDS, Ultra Dry Thermo Scientific, MA, 30 mm^2^ with a resolution of 129 eV) was employed to determine the elemental composition and purity of the nanoparticles, ensuring high copper content and minimal contamination. The microscopy images were analyzed by ImageJ v2 for particle size measurement. The experimental setup was validated through repeated trials, ensuring reproducibility and consistency of results.

### 2.5. Data Analysis and Statistics

Data analysis was conducted using statistical methods. The primary statistical approach involved analysis of variance (ANOVA) to assess the significance of the experimental factors on nanoparticle characteristics. Fisher’s least significant difference (LSD) test was applied for post hoc comparisons to identify specific differences between experimental conditions. Statistical significance was determined at a threshold of *p* < 0.05, ensuring robust conclusions regarding the effects of pH, reducing agent concentration, and residence time. Random seed control was implemented to ensure reproducibility of computational analyses, and validation was achieved through replication of key experiments. The second statistical approach was a two-way ANOVA and the *p*-value was maintained *p* < 0.05 ensuring robust conclusions regarding the effects of flow rate and the stabilizer agent.

## 3. Results

### 3.1. Study of the Physical Characteristics of Microfluidic Devices

The overall length, volume, flow rate, residence times and average velocity of the microfluidic devices for both experiments are reported in Table 3.

Considering the outlet tubes that are 43 cm long and 3 mm in diameter (volume of 3.038 mL), MFR-20 (a and b) has a total volume of 3.11 mL and MFR-40 has a total volume of 3.18 mL.

### 3.2. Fluid Dynamics of Microfluidic Devices in Study

Considering the previous calculations and assuming that the physical properties of the reaction mixture are similar to those of water (ρ = 1000 kg/m^3^ and μ = 1 cP), the Reynolds number (Re) was determined to be 16.7 (see Equation (1)), for both the 20- and 40-turn MFRs in the exploratory experiment. In the advanced experiment, the Re value was 16.7 for a flow rate of 0.4 mL/min and 33.3 when operating at a flow rate of 0.8 mL/min. In all cases evaluated, the obtained values confirm the predominance of a laminar flow regime (Re < 2100).

(1) $$Re=\frac{\rho \cdot v\cdot D_{h}}{\mu }\;$$

On the other hand, Darcy friction factors ($f_{D}$) were determined for laminar flow in square-section conduits (Equation (2)). However, these friction factor values must be adjusted to account for the relative roughness of the FFF-manufactured PETG. Research on the surface quality of components manufactured using FFF has identified layer height as the variable with the greatest impact on PETG roughness. It has been determined that as layer height increases, the roughness of the material increases. Specifically, using a layer height of 0.1 mm, the surface roughness of PETG reaches values of 0.0078 mm and 0.0091 mm when operating at extrusion temperatures of 220 °C and 250 °C (parameters used in this study), respectively [24,25]. To determine the Darcy friction factor ($f_{D}$) and associated pressure drops, an average roughness value derived from the mean of the reported measurements will be used, set at 0.0084 mm.

(2) $$f_{D}=\frac{56.91}{Re}$$

The model by Bahrami et al. [26] allows adjustment of friction factors by accounting for material roughness using the following mathematical expressions for regular roughness:

(3) $$\epsilon =\frac{\epsilon_{o}}{a}$$

where $\epsilon_{o}$ is the roughness of the channel and *a* is the hydraulic radius of the channel. The mathematical expression for calculating the adjusted Darcy friction factor ($f^{*}$) due to the frictional resistance of the material is:

(4) $$f^{*}=f_{D}\;{\cdot R^{*}}_{f}$$

where $f_{D}$ is the Darcy friction factor and ${R^{*}}_{f}$ is the frictional resistance.

At the same time, the frictional resistance depends on the relative roughness of the channel. If the roughness is less than or equal to 0.1, Equation (5) is used, whereas if the roughness is between 0.1 and 0.15, Equation (6) is used:

(5) $$R_{f}^{*}=\frac{1}{1-23\epsilon^{2}}$$

(6) $$R_{f}^{*}=\frac{1}{1-50\epsilon^{2.4}}$$

Therefore, the friction factor for laminar flow in a square pipe, adjusted for the roughness of the material, is calculated by substituting Equation (2) into Equation (4), using the following mathematical expression:

(7) $$f^{*}=\frac{56.91}{Re}{\cdot R^{*}}_{f}$$

Finally, substituting Equation (7) into the Equation of the pressure drop for laminar flow gives the following equation:

(8) $$\Delta P=\left(\frac{56.91}{Re}{\cdot R^{*}}_{f}\right)\cdot \left(\frac{L}{D_{h}}\right)\cdot \left(\frac{\rho \cdot v^{2}}{2}\right)$$

All these calculations are summarized in Table 4. Considering that each NE-300 syringe pump can exert a maximum force of 155.7 N according to its technical data sheet, and the base of each syringe has a diameter of 2.67 cm, each pump can exert an approximate pressure of 278 mPa. Therefore, the system did not experience pressure issues. For calculation purposes, minor pressure losses associated with the micromixers, coil bends, reducers, and outlet connections were considered negligible, assuming that the greatest resistance is exerted by the main microchannels.

Another important parameter is the Dean number (De). For the 20- and 40-turn MFRs in the exploratory experiment, De was 9.6. In the advanced experiment, De was 9.6 for a flow rate of 0.4 mL/min and 19.2 at a flow rate of 0.8 mL/min. These values, which are close to and greater than 10, allow the formation of stable Dean vortices that facilitate the mixing of the reactive substances. To calculate the Peclet number (Pe), the monoascorbate anion (C_6_H_7_O_6_^−^) and the copper (II) ion in aqueous solution were considered at experimental concentrations (0.1 M and 0.5 M, respectively) to determine the species with the lower diffusivity coefficient.

Because the ascorbic acid solution was adjusted to pH 8, the predominant chemical species in the system is the monoascorbate anion. This prevalence is due to the fact that the pH of the reducing solution lies between the values of the acid dissociation constants, pK_a1_ 4.17 and pK_a2_ 11.57, corresponding to the transitions of the monoascorbate anion and diascorbate anion (C_6_H_6_O_6_), respectively [4]. Based on this consideration, the diffusivity of ascorbic acid in aqueous solution at a concentration of 0.5 M was used as a reference, with a value of 7.5 × 10^−10^ m^2^/s. A correction factor of 77% was applied to this value due to ion–solvent interaction, resulting in a diffusivity coefficient of 5.8 × 10^−10^ m^2^/s at 0.5 M in aqueous medium for the monoascorbate anion [27]. The presence of the monoascorbate anion in the reducing solution is evident by its bright yellow color obtained at the end of the reaction. On the other hand, it was found that the diffusivity coefficient of the copper (II) ion in aqueous medium at a concentration of 0.1 M reaches a value of 5.6 × 10^−10^ m^2^/s [28], a value very similar to the diffusivity of the monoascorbate anion; however, since it is lower, the copper (II) ion is the species that limits diffusion in the solution.

Finally, the Peclet number (Pe) was determined for the 20- and 40-turn MFRs in the exploratory experiment, yielding a value of 29,551. In the advanced experiment, the Pe value was 29,551 for a flow rate of 0.4 mL/min and 51,910 at a flow rate of 0.8 mL/min.

The high Peclet numbers indicate that the system operates in a regime where advection dominates molecular diffusion by more than four orders of magnitude. This condition, which in straight microchannels would result in reactant segregation and inefficient mixing, is compensated for by the serpentine architecture of the MFR microchannels. The generation of secondary flows reduces the diffusion length scale, allowing nanoparticle synthesis to occur homogeneously.

### 3.3. Quantification of the Copper Conversion

The copper conversion in MFR-20 was quantified by electrical conductivity measurement at the different residence times, by following a calibration curve established as:

(9) $$\sigma \;=\;25.806\;x^{2}\;+\;8.3383\;x\;+\;9.7377\;$$

where σ is solution electrical conductivity and x is copper conversion.

The R-squared value was 0.9967. Table 5 presented the copper conversion determined with this method at the room temperature of the laboratory (16 °C).

Assuming that the MFR behaves as a plug flow reactor (PFR), the correlation between residence time and conversion is established by the following mathematical expression:

(10) $${\tau =C}_{A_{0}}\int_{0}^{x}\frac{dx}{-r_{A}}\;$$

where τ corresponds to the residence time, $C_{A_{0}}$ is the initial concentration of copper, x is the copper conversion and $r_{A}$ is the reaction rate.

Given that the concentration of the excess reactant remains constant along the MFR, it can be assumed that the reaction rate is governed by a pseudo-first-order kinetic model:

(11) $$-r_{A}=k^{\prime }C_{A_{0}}\left(1-x\right)$$

Thus, Equation (10) becomes the following mathematical expression:

(12) $${\tau =C}_{A_{0}}\int_{0}^{x}\frac{dx}{k^{\prime }\cdot C_{A_{0}}\left(1-x\right)}$$

After integration, Equation (11) becomes the following mathematical expression:

(13) $$k^{\prime }\tau =-ln\;\left(1-x\right)$$

Therefore, based on the data in Table 5 and Equation (13), the pseudo-first-order kinetic constant for the estimated conversions as a function of residence time was determined to be 0.0344 min^−1^. Furthermore, considering the maximum conversion (test E4), the final copper concentration is 0.0364 M yielding a reaction rate of 0.00125 M/min.

Finally, the mathematical expression that model the conversion of MFR-20 as a function of residence time corresponds to Equation (14):

(14) $$x\;\left(\tau \right)=1-e^{-0.0344\;\tau }$$

### 3.4. Results from the Exploration Experiment

After conducting the exploration experimental tests described in the methodology, applying the synthesis process and data collection methods, the results of this stage were tabulated in Table 6.

#### 3.4.1. Effects on Morphology of the Copper Nanoparticles

**Figure 2:**
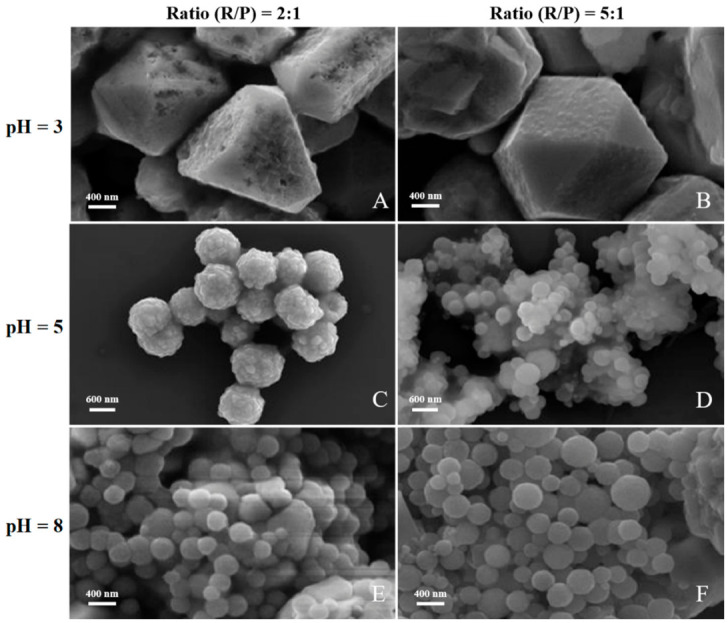
SEM images of CuNPs obtained in the exploration stage at 50,000× for MFR-20a. The effect of pH (3, 5 and 8) at a ratio R/P of 2:1 (**A**,**C**,**E**) and a ratio R/P of 5:1 (**B**,**D**,**F**) on morphology is observed.

The particles obtained with MFR-20a are presented in Figure 2. It is possible to see that images A and B show nanoparticles with well-defined polyhedral morphologies. In image C, spherical nanostructures composed of coalesced polyhedral sub-units are identified. Image D reveals a similar aggregation pattern, but with a greater number of constituent spherical particles. Images E and F show a clear transition toward predominantly and uniformly spherical CuNPs, especially in image F, where the spherical morphology is clear and homogeneous.

**Figure 3:**
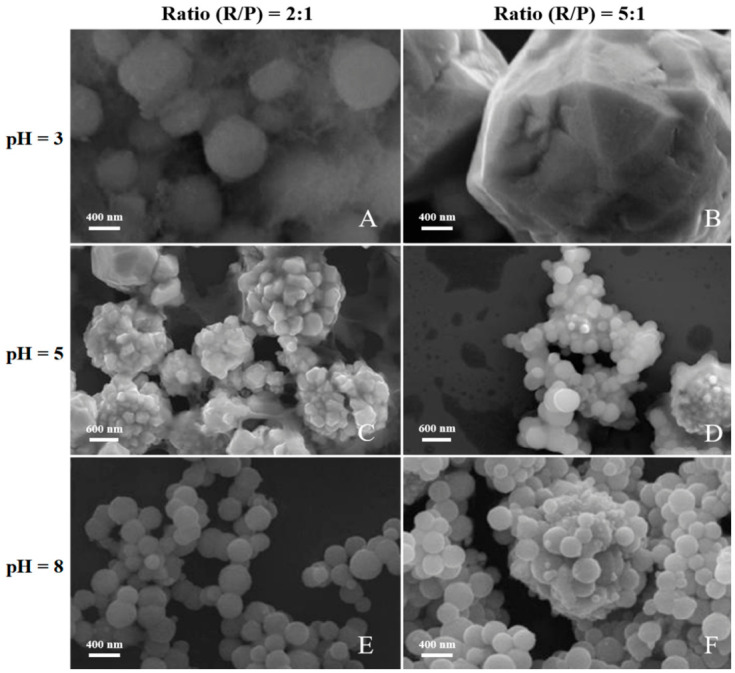
SEM images of CuNPs obtained in the exploration stage at 50,000× for MFR-40. The effect of pH (3, 5 and 8) at a ratio (R/P) of 2:1 (**A**,**C**,**E**) and a ratio (R/P) of 5:1 (**B**,**D**,**F**) on morphology is observed.

In Figure 3, it is possible to observe the images of the particles obtained with MFR-40. Images A and B show nanoparticles with a mixed morphology of irregular spheres and massive polyhedral structures. In Figure 3C, semispherical nanostructures composed of coalesced polyhedral subunits are identified. In Figure 3D reveals a similar aggregation pattern, but with a greater number of constituent spherical particles. Figure 3E,F show a clear transition toward predominantly and uniformly spherical CuNPs, with image F highlighting the presence of large globular agglomerates.

#### 3.4.2. Effect on Particle Size for the Exploratory Study

Table 7 presents the statistical results of the ANOVA performed for the size (average diameters) of copper nanoparticles, detailing the impact of the main effects of the individual parameters and their interactions.

It is observed that the pH factor exerts a critical and statistically significant effect (*p* < 0.001), accounting for the largest proportion of the variability in the system. Likewise, the ratio (R/P) proved to be a significant factor (*p* = 0.031), while the number of MFR turns did not show a direct influence on statistical significance (*p* = 0.139). Upon analyzing the interactions, the two-way interaction term (R/P) × pH yielded a statistically significant effect (*p* = 0.006). Finally, the three-way interaction (R/P) × pH × MFR turns were found to have a highly significant impact on the average particle diameter (*p* = 0.001), unlike the second-order interactions involving the number of turns (*p* > 0.05).

#### 3.4.3. Effect on the Particle Homogeneity

It is observed (see Table 8) that the pH factor does not exert a statistically significant effect (*p* = 0.673), accounting for a smaller proportion of the system’s variability. Likewise, the ratio (R/P) was not a significant factor (*p* = 0.637), while the number of MFR turns also did not show a direct influence with statistical significance (*p* = 0.726). Upon analyzing the interactions, the two-way interaction (R/P) × pH yielded a non-significant effect (*p* = 0.654). Finally, the three-way interaction (R/P) × pH × MFR turns was found to have a non-significant impact on particle homogeneity (*p* = 0.812), unlike what was observed for CuNP size.

Since the pH factor was shown to have a statistically significant effect on the size of the copper nanoparticles (*p* < 0.001) and given the significance of the three-way interaction between the R/P ratio, pH, and MFR turns (*p* = 0.001), it was decided to adjust the alkalinity of the reducing solution to a pH of 8. This condition not only favors the production of smaller CuNPs but also ensures the prevalence of the monoascorbate anion, whose pKa ranges from 4.17 to 11.57, and further increases the total reduction potential of the reaction. Additionally, this alkaline condition allowed for the formation of highly spherical morphology.

On the other hand, the number of MFR turns did not show a significant impact (*p* = 0.139); therefore, it was decided to conduct the advanced experiment using MFR-20, thereby saving costs during manufacturing. Finally, a ratio (R/P) of 5 was established to ensure sufficient electron availability to promote the chemical reduction of copper ions.

### 3.5. Advanced Experiment

Taking the exploratory results into account, the advanced experimental tests outlined in the methodology were carried out. By applying the synthesis process and data collection methods, the results of this stage were tabulated in Table 9.

#### 3.5.1. Effect on Morphology of Copper Particles

**Figure 4:**
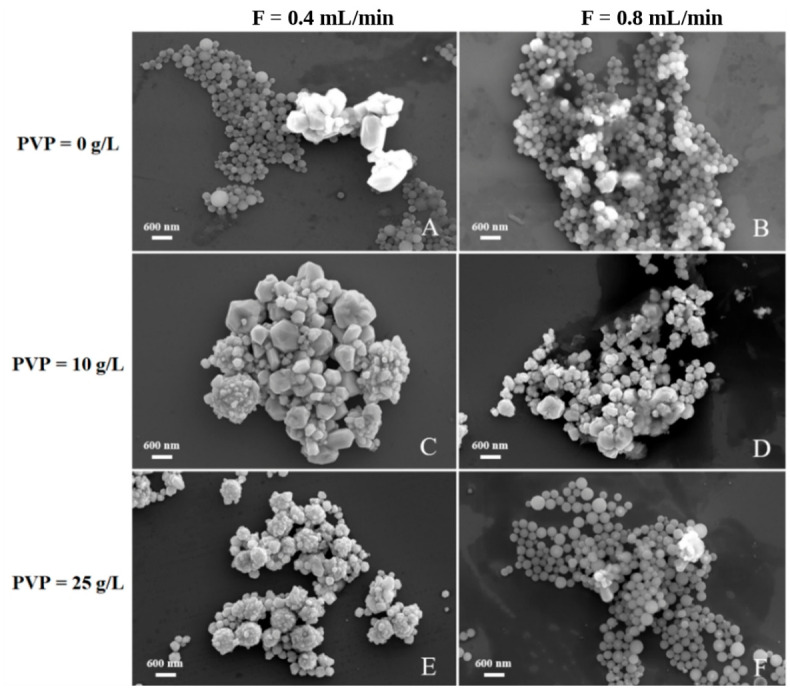
SEM images of the CuNPs obtained in the advanced stage at 25,000× using MFR-20b. The effect of the PVP additive (0, 10 and 25 g/L) at a total chemical reagent flow rate of 0.4 mL/min (**A**,**C**,**E**) and 0.8 mL/min (**B**,**D**,**F**) on morphology is observed.

Figure 4 presents a series of FESEM images at 25,000×, revealing the morphological evolution of CuNPs following the synthesis under specific experimental conditions of the advanced experiment parameters.

Figure 4A,B show CuNPs with spherical morphologies along with larger polyhedral agglomerates. In Figure 4C, nanostructured CuNPs composed of agglomerated polyhedral particles are identified. Figure 4D reveals a similar aggregation pattern, with smaller subunits. Figure 4E,F show a transition toward structures with a lower degree of compaction; Figure 4E highlights the presence of nanostructures with a hemispherical morphology composed of polyhedral subunits, while Figure 4F shows a predominantly spherical, organized, and uniform population.

#### 3.5.2. Effect on Particle Size

Statistical results of the ANOVA for the size (average diameters) of CuNPs are presented in Table 10, detailing individually the impact of the main effects and their higher-order interactions.

It is observed that the PVP additive factor exerts a critical and statistically significant effect (*p* < 0.001), accounting for the largest proportion of the system’s variability. Likewise, flow rate was found to be a significant factor (*p* = 0.014). Upon analyzing the interactions, the two-way interaction between flow rate and the PVP additive yielded a statistically significant effect (*p* = 0.009).

#### 3.5.3. Effect on Homogeneity of the Particle Size

Table 11 presents the statistical results of the ANOVA for the homogeneity (mean standard deviation) of CuNPs, detailing individually the impact of the main effects and their higher-order interactions.

It is observed that the PVP additive factor exerts a critical and statistically significant effect (*p* = 0.011), accounting for the largest proportion of the system’s variability. In contrast, flow was not found to be a significant factor (*p* = 0.115). Upon analyzing the interactions, the two-way interaction between flow and the PVP additive yielded a statistically insignificant effect (*p* = 0.446), with this interaction remaining above the established threshold (*p* > 0.05).

#### 3.5.4. Effect on Copper Nanoparticle Purity

Table 12 presents the statistical results of the ANOVA for the purity (average copper composition) of the CuNPs, detailing individually the impacts of the main effects and their higher-order interactions.

It is observed that under the evaluated experimental conditions and considering the established confidence level, none of the main effects, flow (*p* = 0.076) and the PVP additive (*p* = 0.551), nor their two-way interactions, flow × PVP additive (*p* = 0.268), reached statistical significance (*p* > 0.05). The intrinsic random variability of the process, measured by the pure error of the replicates, accounted for a considerable fraction of the total observed variation, exceeding the effect of the controlled factors.

## 4. Discussion

The statistical analysis of this study allowed the understanding of the mechanisms governing the synthesis of copper nanoparticles in microfluidic reactors fabricated by 3D printing with a thermoplastic filament and the comparison of these results with the scientific literature reviewed.

### 4.1. Effects of Chemical Parameters on Copper Nanoparticles

The fact that the ratio (R/P) affects the reduction in the particle size is consistent with the findings reported by Xu et al. [12], who determined that an increase in the concentration of the reducing agent relative to the copper precursor causes a decrease in the average diameter and homogeneity of the CuNPs, a behavior also observed in batch systems studied by Liu et al. [29]. From a chemical standpoint, an excess of available electrons promotes the continuous chemical reduction of copper ions, increasing its concentration, according to the LaMer and Dinegar model, until reaching the critical point where the nucleation stage occurs. Then, it begins to decrease slowly, consuming the precursor and gradually limiting the growth of the copper nanoparticles. However, in this study, the ratio (R/P) by itself proved to be insignificant for this variable.

On the other hand, pH emerged as an important parameter for obtaining the spherical morphologies and small sizes of CuNPs in the system studied. In the literature, Liang et al. [17] and Gande et al. [30] used ascorbic acid as a reducing agent at a basic pH to increase the amount of the monoascorbate anion (C_6_H_7_O_6_^−^) in the reaction, which releases the electrons necessary to reduce copper ions. Also, from a thermodynamic standpoint, increasing the pH of the reducing solution increases the electrochemical reduction potential of the oxidation half-reaction and thereby the total reduction potential, further favoring the chemical reaction and promoting the formation of spherical nuclei.

The addition of a stabilizer as PVP proved to be the absolute governing parameter for morphology and particle size of the CuNPs. The phenomenon is explained by the interaction between the pyrrolidone rings and the surface of copper atoms preventing the agglomeration and growth of the copper nanoparticles. This behavior is consistent with the findings reported by Ramprasad et al. [10], Xu et al. [11], and Gande et al. [30], who highlight the superiority of PVP as an additive in improving the homogeneity of CuNPs in MFR. In contrast, increasing PVP concentration did not exert a statistically significant effect on CuNP purity, demonstrating that while the polymeric surfactant effectively inhibits particle agglomeration, it does not completely halt surface oxidation. Copper oxidation at the nanoscale remains a highly spontaneous, time-dependent thermodynamic phenomenon; hence, the gradual decline in elemental purity is directly attributed to the storage intervals elapsed prior to analysis. Crucially, these storage windows varied depending on the operational scheduling and baseline queues at the centralized characterization facilities, allowing ambient oxygen diffusion to partially compromise the metallic cores despite the presence of the polymeric stabilizer.

### 4.2. Effects of Hydrodynamic Parameters on Copper Nanoparticles

Variations in the number of MFR turns (20 and 40) had no effect on the morphology and particle size of the copper nanoparticles obtained. This is explained by the operating conditions where laminar flow (Re = 16.7) with induced Dean vortices (De = 9.6) and advective mixing (Pe = 29,551) occurs. Copper nanoparticle formation takes places upon entry into the third turning channel of the MFR. Since the three stages of CuNP formation (supersaturation, nucleation, and growth) are spatially completed within the third turning channel of the MFR, doubling the reactor length from 20 to 40 only extends the steady-state transit zone where no significant changes occur. This finding is consistent with Zhang et al. [31] who found that the size and homogeneity of copper nanoparticles become insensitive to the design once the minimum residence time required for chemical reduction is ensured.

From the perspective of transport phenomena, an increase in total flow rate inversely reduces the residence time within the MFR. This reduced time interrupts the growth stage of copper nuclei, slowing the deposition of atoms onto pre-existing nuclei. Consequently, the increase in flow rate directly contributes to the production of CuNPs with smaller sizes while maintaining spherical morphology, confirming the trend reported by Liang et al. [17], who obtained smaller sizes by increasing the flow rate. However, this contradicts the observations of Wei et al. [32] and Xu et al. [11], who associated an increased flow rate with larger copper nanoparticle sizes. This could be due to differences in the designs of the MFRs they used, which were helical and T-type, respectively.

To overcome the inherent limitations of theoretical hydrodynamic modeling, future investigations must incorporate Computational Fluid Dynamics (CFD) to provide a high-resolution spatial and temporal map of the reaction environment. While this study assumes a near-ideal plug flow reactor (PFR) behavior supported by stable Dean vortices and laminar regimes, numerical simulations, as demonstrated by Song et al. [15] and Biswas et al. [16], would reveal the impact of flow fluctuations and the non-ideal RTD tailing. This phenomenon is primarily caused by the no-slip boundary condition at the channel walls and the inherent mechanical limitations of syringe pumps, which can extend the time required for the system to reach a steady state. Such computational insights are critical to confirm whether the completion of supersaturation, nucleation, and growth stages is indeed spatially confined within the initial mixing turns or if axial dispersion contributes to the observed polydispersity. Furthermore, CFD-assisted design could optimize the throughput-to-pressure drop ratio, enhancing the scalability of this sustainable 3D-printed platform while ensuring the reproducible synthesis of CuNPs with atomic precision.

### 4.3. Reaction Kinetics and Copper Conversion

Based on the experimental data obtained from the MFR-20, the pseudo-first-order kinetic constant of 0.0344 min^−1^ and a reaction rate of 0.00125 M/min were determined. This confirms the high temporal performance of the microfluidic system. Upon analyzing the conversion profile as a function of residence time, it was observed that a decrease in time results in an increase in system conversion (from 14.6% to 27.2% for flow rates between 0.4 and 1.0 mL/min, respectively). This behavior confirms that the hydrodynamics of continuous flow optimize mass transfer and synthesis rate. This efficient mass transfer and the synthesis rates obtained are consistent with the findings reported by Zhang et al. [31], who highlighted that the microfluidic synthesis of CuNPs allows for a drastic reduction in traditional chemical synthesis times, from 10 min in batch to just 30 s in continuous flow, emphasizing the need to operate at optimal residence times that ensure the nucleation and growth stages.

Likewise, the kinetic results are consistent with the research by Gande et al. [30], who, using a helical MFR, demonstrated synthesis rates superior to the batch method, achieving stable copper nanoparticles in just 1 min of operation. Similarly, the increase in conversion at shorter residence times observed in Table 4 validates the approach of Biswas et al. [16] regarding the importance of operating under controlled residence times by adjusting the injection flow rate to maximize the production yield of the system. Finally, the mathematical expression obtained to model conversion (Equation (14)) confirms the viability of these microreactors for intelligently predicting, controlling, and scaling the continuous production of CuNPs.

## 5. Conclusions

In this work, the feasibility of the sustainable synthesis of copper nanoparticles using 3D-printed microfluidic devices fabricated via the fused filament technique with glycol-modified polyethylene terephthalate was successfully demonstrated. Through a systematic experimental design, pH was determined to be the most significant factor affecting the physicochemical properties of the system, establishing that an alkaline medium of pH, combined with a reductant-to-precursor ratio of 5:1, favors the production of copper nanoparticles with highly spherical morphologies and significantly smaller diameters. From an analytical and fluid dynamics perspective, modeling the serpentine architecture of the devices evidenced the formation of stable Dean vortices within a dominant laminar flow regime, which compensated for the predominance of advection over molecular diffusion and facilitated homogeneous mixing of the reagents. Furthermore, statistical evaluation showed that the number of turns in the mixer did not exert a direct impact on the final particle size, consolidating the 20-turn device as an efficient and lower-cost alternative for nanomaterial manufacturing. These results validate the use of additive manufacturing and continuous microfluidics as a robust, economical, and low-environmental-impact platform for the controlled and reproducible production of metallic nanoparticles with potential industrial scalability.

## Figures and Tables

**Table 1:** Main parameters in the study for the first DoE applied.

Parameter	Values		
Ratio (R/P)	2:1		5:1
MFR Turns	20		40
pH	3	5	8

**Table 2:** Main parameters in the study for the second DoE applied.

Parameter	Values		
Flow rate (mL/min)	20		40
PVP (g/L)	0	10	25

**Table 3:** Length, volume, flow rates, residence times and average velocity of MFRs.

Microfluidic Devices	Length MFR (m)	Volume MFR (mL)	Flow Rate (mL/min)	Residence Time (s)	Average Velocity (m/s)
MFR-20a	0.463	0.0740	0.4	11.12	0.0417
MFR-40	0.917	0.1470	0.4	22.24	0.0417
MFR-20b	0.463	0.0740	0.4	11.12	0.0417
			0.8	5.56	0.0833

**Table 4:** Reynolds numbers, Darcy friction factors, and pressure drop of the MFRs in both experiments.

Microfluidic Devices	Flow (mL/min)	Re	*f_D_*	f*	ΔP (Pa)
MFR-20a	0.4	16.7	3.41	3.46	3456.9
MFR-40	0.4	16.7	3.41	3.46	6482.9
MFR-20b	0.4	16.7	3.41	3.46	3456.9
	0.8	33.3	1.71	1.73	6913.8

**Table 5:** Residence times and conversions of MFR-20 (3.11 mL).

Test	2F (mL/min)	τ (min)	σ at 16 °C (μS/cm)	Conversion (x)	(−1)⋅ln(1 − x)
E1	0.40	7.78	11.25	0.146	0.157
E2	0.60	5.19	12.44	0.238	0.272
E3	0.80	3.89	12.70	0.258	0.298
E4	1.00	3.11	12.89	0.272	0.317

**Table 6:** Results of exploration experiment.

	Independent Variables			Dependent Variables		
Test	Ratio (R/P)	pH	Turn MFR	Morphology	Diameter. (nm)	Std. Dev. (nm)
P1	2	3	20	0	2233.2	127.6
P2	2	5	20	2	655.1	85.1
P3	2	5	20	2	498.5	209.3
P4	2	8	20	2	339.9	72.0
P5	5	3	20	0	2811.6	280.4
P6	5	5	20	2	413.2	172.6
P7	5	5	20	1	238.1	127.3
P8	5	8	20	1	350.0	125.3
P9	2	3	40	1	850.2	237.9
P10	2	5	40	2	452.6	404.9
P11	2	5	40	2	1116.3	364.7
P12	2	8	40	1	354.7	95.1
P13	5	3	40	2	5195.9	115.7
P14	5	5	40	1	402.5	167.2
P15	5	5	40	1	280.0	107.1
P16	5	8	40	1	288.7	122.9

**Table 7:** Analysis of Variance (ANOVA) for the CuNP size exploratory study.

Independent Variables and Intersections	Sum of Squares (SS)	Degrees of Freedom (DF)	Mean Square (MS)	F	*p*	Significance (α = 0.05)
Ratio (R/P)	684,711.1	1	684,711.1	10.7	0.031	Significant
pH	16,279,178.5	2	8,139,589.3	127.5	<0.001	Highly Significant
MFR Turns	216,912.5	1	216,912.5	3.4	0.139	Non-Significant
Ratio (R/P) × pH	3,381,440.2	2	1,690,720.1	26.5	0.006	Significant
Ratio (R/P) × MFR Turns	94,812.1	1	94,812.1	1.5	0.290	Non-Significant
pH × MFR Turns	231,451.1	2	115,725.6	1.8	0.275	Non-Significant
Ratio (R/P) × pH × MFR Turns	8,103,756.6	2	4,051,878.3	63.5	0.001	Highly Significant
Pure Error	255,340.3	4	63,835.1			
Total	29,247,602.5	15				

**Table 8:** Analysis of Variance (ANOVA) for the homogeneity of the CuNPs.

Independent Variables and Interactions	Sum of Squares (SS)	Degrees of Freedom (DF)	Mean Square (MS)	F	*p*	Significance (α = 0.05)
Ratio (R/P)	441.0	1	441.0	0.3	0.637	Non-Significant
pH	1486.4	2	743.2	0.4	0.673	Non-Significant
MFR Turns	240.3	1	240.3	0.1	0.726	Non-Significant
Ratio (R/P) × pH	894.8	2	447.4	0.3	0.654	Non-Significant
Ratio (R/P) × MFR Turns	312.1	1	312.1	0.2	0.691	Non-Significant
pH × MFR Turns	1522.3	2	761.2	0.4	0.652	Non-Significant
Ratio (R/P) × pH × MFR Turns	2007.6	2	1003.8	0.6	0.812	Non-Significant
Pure Error	6776.5	4	1694.1			
Total	13,681.0	15				

**Table 9:** Results of advanced experiment.

	Independent Variables		Dependent Variables			
Test	Flow Rate (mL/min)	PVP (g/L)	Morphology	Diameter (nm)	Std. Dev. (nm)	Cu Purity (Weight %)
P1	0.4	0	2	369.6	186.3	55.6
P2	0.4	0	1	285.7	102.3	44.6
P3	0.4	0	2	446.6	280.4	90.8
P4	0.4	10	0	589.8	412.6	93.8
P5	0.4	10	0	634.7	430.3	93.9
P6	0.4	10	0	542.4	352.6	92.5
P7	0.4	25	0	400.0	272.9	96.8
P8	0.4	25	1	414.8	109.8	38.4
P9	0.4	25	2	558.9	314.1	93.9
P10	0.8	0	2	253.0	63.0	47.2
P11	0.8	0	2	313.7	86.3	37.7
P12	0.8	0	1	359.2	93.1	51.0
P13	0.8	10	2	359.7	166.3	76.0
P14	0.8	10	0	236.7	124.1	69.9
P15	0.8	10	0	375.2	270.9	93.1
P16	0.8	25	2	371.4	142.2	33.4
P17	0.8	25	1	413.2	140.8	36.7
P18	0.8	25	2	467.3	185.3	73.6

**Table 10:** Analysis of Variance (ANOVA) for CuNP size.

Independent Variables and Interactions	Sum of Squares (SS)	Degrees of Freedom (DF)	Mean Square (MS)	F	*p*	Significance (α = 0.05)
Flow rate	59,316.1	1	59,316.1	8.6	0.014	Significant
PVP	162,540.4	2	81,270.2	11.8	<0.001	Highly Significant
Flow rate × PVP	101,912.8	2	50,956.4	7.4	0.009	Significant
Pure Error	82,725.5	12	6893.8	—	—	—
Total	406,494.8	17	—	—	—	—

**Table 11:** Analysis of variance (ANOVA) for the homogeneity of CuNPs.

Independent Variables and Interactions	Sum of Squares (SS)	Degrees of Freedom (DF)	Mean Square (MS)	F	*p*	Significance (α = 0.05)
Flow Rate	26,064.2	1	26,064.0	2.9	0.115	Non-Significant
PVP	112,108.1	2	56,054.0	6.3	0.011	Significant
Flow Rate × PVP	15,462.4	2	7731.2	0.9	0.446	Non-Significant
Pure Error	107,147.7	12	8929.0			
Total	260,782.4	17				

**Table 12:** Analysis of Variance (ANOVA) for the purity of CuNPs.

Independent Variables and Interactions	Sum of Squares (SS)	Degrees of Freedom (DF)	Mean Square (MS)	F	*p*	Significance (α = 0.05)
Flow Rate	2153.4	1	2153.4	3.7	0.076	Non-Significant
PVP	711.8	2	355.9	0.6	0.551	Non-Significant
Flow Rate × PVP	1684.3	2	842.2	1.4	0.268	Non-Significant
Pure Error	6994.1	12	582.8			
Total	11,543.6	17				

## Data Availability

The original contributions presented in this study are included in the article. Further inquiries can be directed to the corresponding author.

## Abbreviations

The following abbreviations are used in this manuscript:

CuNPs	Copper nanoparticles
MFR	Microfluid reactor
FFF	Fused Filament Fabrication
PETG	Polyethylene Terephthalate Glycol
AM	Additive Manufacturing
MNPs	Metal nanoparticles

## References

[1] Nguyen T.D., Ngo S.T., Hoang Y.H., Thai N.T.T., Nguyen H.T.T., Trinh G.T.N. (2025). Studying the synthesis, antimicrobial activity, and phenol red removal of gelatin-stabilized copper nanoparticles. Nanoscale Adv..

[2] Akintelu S.A., Oyebamiji A.K., Olugbeko S.C., Latona D.F. (2021). Green chemistry approach towards the synthesis of copper nanoparticles and its potential applications as therapeutic agents and environmental control. Curr. Res. Green Sustain. Chem..

[3] Dinda G., Halder D., Vazquez-Vazquez C., Lopez-Quintela M.A., Mitra A. (2015). Green Synthesis of Copper Nanoparticles and their Antibacterial Property. J. Surf. Sci. Technol..

[4] Benabbas A., Breyton G., Especel C., Le Valant A., Ricolleau C., Wang G., Mineva T., Nelayah J., Guesmi H., Epron F. (2025). Facile and green synthesis of monodisperse sub-10 nm copper and tin nanoparticles using l-ascorbic acid as the reducing agent. RSC Adv..

[5] Umer A., Naveed S., Ramzan N., Rafique M.S. (2012). Selection of a Suitable Method for the Synthesis of Copper Nanoparticles. Nano.

[6] Wu C., Mosher B.P., Zeng T. (2006). One-step green route to narrowly dispersed copper nanocrystals. J. Nanopart. Res..

[7] Dang T.M.D., Le T.T.T., Fribourg-Blanc E., Dang M.C. (2011). Synthesis and optical properties of copper nanoparticles prepared by a chemical reduction method. Adv. Nat. Sci. Nanosci. Nanotechnol..

[8] Bezelya A., Küçüktürkmen B., Bozkır A. (2023). Microfluidic Devices for Precision Nanoparticle Production. Micro.

[9] Ke T., Le Y., Wang J.-X., Chu G.-W., Chen J.-F., Shao L. (2010). Cu nanoparticle preparation in a tube-in-tube microchannel reactor and encapsulation by silica. Mater. Lett..

[10] Ramprasad S., Ramsing P.E., Miller R.T., Rundel J.T., Remcho V.T., Palo D.R. (2011). Copper Nanoparticle Synthesis in Continuous mode by the Polyol method—Progress towards Nanomanufacturing. 2011 11th IEEE International Conference on Nanotechnology.

[11] Xu L., Peng J., Srinivasakannan C., Zhang L., Zhang D., Liu C., Wang S., Shen A.Q. (2014). Synthesis of copper nanoparticles by a T-shaped microfluidic device. RSC Adv..

[12] Xu L., Peng J., Srinivasakannan C., Chen G., Shen A.Q. (2015). Synthesis of copper nanocolloids using a continuous flow based microreactor. Appl. Surf. Sci..

[13] Xu L., Srinivasakannan C., Peng J., Yan M., Zhang D., Zhang L. (2015). Microfluidic reactor synthesis and photocatalytic behavior of Cu@Cu_2_O nanocomposite. Appl. Surf. Sci..

[14] Xu L., Srinivasakannan C., Peng J., Zhang L., Zhang D. (2017). Synthesis of Cu-CuO nanocomposite in microreactor and its application to photocatalytic degradation. J. Alloys Compd..

[15] Song Y., Li R., Sun Q., Jin P. (2011). Controlled growth of Cu nanoparticles by a tubular microfluidic reactor. Chem. Eng. J..

[16] Biswas S., Miller J.T., Li Y., Nandakumar K., Kumar C.S.S.R. (2012). Developing a Millifluidic Platform for the Synthesis of Ultrasmall Nanoclusters: Ultrasmall Copper Nanoclusters as a Case Study. Small.

[17] Liang Y., Shinozaki Y., Yagyu H. (2018). Synthesis of Copper Nanoparticles Using Glass Microfluidic Device. Proceedings.

[18] Garcia-Cardosa M., Granados-Ortiz F.-J., Ortega-Casanova J. (2021). A Review on Additive Manufacturing of Micromixing Devices. Micromachines.

[19] Amreen K., Goel S. (2021). Review—Miniaturized and Microfluidic Devices for Automated Nanoparticle Synthesis. ECS J. Solid State Sci. Technol..

[20] Kitson P.J., Rosnes M.H., Sans V., Dragone V., Cronin L. (2012). Configurable 3D-Printed millifluidic and microfluidic ‘lab on a chip’ reactionware devices. Lab Chip.

[21] Bressan L.P., Robles-Najar J., Adamo C.B., Quero R.F., Costa B.M., de Jesus D.P., da Silva J.A. (2019). 3D-printed microfluidic device for the synthesis of silver and gold nanoparticles. Microchem. J..

[22] Bressan L.P., Lima T.M., Da Silveira G.D., Da Silva J.A.F. (2020). Low-cost and simple FDM-based 3D-printed microfluidic device for the synthesis of metallic core–shell nanoparticles. SN Appl. Sci..

[23] Zhang Y., Li M., Tseng T.-M., Schlichtmann U. (2024). Open-source interactive design platform for 3D-printed microfluidic devices. Commun. Eng..

[24] Taqdissillah D., Muttaqin A.Z., Darsin M., Dwilaksana D., Ilminnafik N. (2022). The Effect of Nozzle Temperature, Infill Geometry, Layer Height and Fan Speed on Roughness Surface in PETG Filament. J. Mech. Eng. Sci. Technol..

[25] Djurovic S., Velikinac N., Ivkovic M., Lazarević D., Mišić M., Stojčetović B., Petković M. (2025). Influence of layer height on the surface roughness of FDM printed PETG parts. Conference Proceedings: 60th Anniversary of the Association of Production Engineering of Serbia.

[26] Bahrami M., Yovanovich M.M., Culham J.R. (2006). Pressure Drop of Fully-Developed Laminar Flow in Rough Microtubes. J. Fluids Eng..

[27] Shamim M., Baki S. (1980). Diffusion measurements in aqueous L-ascorbic acid solutions. Aust. J. Chem..

[28] Emanuel A., Olander D.R. (1963). Diffusion Coefficients of Copper Sulfate in Water and Water in n-Butyl Alcohol. J. Chem. Eng. Data.

[29] Liu Q., Zhou D., Yamamoto Y., Ichino R., Okido M. (2012). Preparation of Cu nanoparticles with NaBH_4_ by aqueous reduction method. Trans. Nonferrous Met. Soc. China.

[30] Gande V.V., Podupu P.K.R., Berry B., Nere N.K., Pushpavanam S., Singh M.R. (2024). Engineering advancements in microfluidic systems for enhanced mixing at low Reynolds numbers. Biomicrofluidics.

[31] Zhang Y., Jiang W., Wang L. (2010). Microfluidic synthesis of copper nanofluids. Microfluid. Nanofluidics.

[32] Wei X., Wang L. (2010). Synthesis and thermal conductivity of microfluidic copper nanofluids. Particuology.

